# Gut microbiome profile of Chinese hypertension patients with and without type 2 diabetes mellitus

**DOI:** 10.1186/s12866-023-02967-x

**Published:** 2023-09-09

**Authors:** Hongying Ding, Yue Xu, Yinhong Cheng, Haoliang Zhou, Shiye Dong, Jian Wu, Jin Lv, Xiaosheng Hu, Oushan Tang

**Affiliations:** 1 Department of Cardiovascular Medicine, Shaoxing Second hospital, Shaoxing, Zhejiang China; 2 Shanghai Biotecan Pharmaceuticals Co., Ltd, Shanghai, China; 3 Shanghai Zhangjiang Institute of Medical Innovation, Shanghai, China; 4 The First Affiliated Hospital of Medical College of Zhejiang University, Hangzhou, China

**Keywords:** Gut microbiota, Hypertension, Type 2 diabetes mellitus, Comorbidity, 16S rRNA gene sequencing

## Abstract

**Background:**

The coexistence of hypertension and type 2 diabetes mellitus (T2DM) may largely increase the risk for cardiovascular disease. However, there is no clear consensus on the association between hypertension and the risk of diabetes. Gut microbiota plays important roles in the development of hypertension and T2DM, but whether there is difference between hypertension patients with or without T2DM has not been explored yet.

**Methods:**

We recruited 101 hypertension patients in this study (72 patients without T2DM named HT group and 29 patients with T2DM named HT-T2DM group). Their blood samples were collected for testing clinical characteristics and fecal samples were tested for bacterial DNA using 16 S ribosomal RNA gene sequencing targeting the V3 and V4 region. The data of 40 samples were downloaded from project PRJNA815750 as health control (HC group) in this study. The community composition and structure of the microbiome, taxonomic difference, co-occurrence network and functional enrichment were analyzed by alpha/beta diversity, LEfSe, Fruchterman Reingold’s algorithm and PICRUSt2 functional analysis, respectively.

**Results:**

Alpha and beta diversity analysis showed significant differences in microbial community richness and composition among the three groups. The HC group had a significantly higher Simpson index and a distinct microbiota community compared to the HT and HT-T2DM groups, as demonstrated by significant differences in unweighted and weighted UniFrac distances. The LEfSe analysis identified specific taxa that had significantly different abundance among the groups, such as *Bacteroides uniformis*, *Blautia wexlerae*, *Alistipes putredinis*, and *Prevotella stercorea* in the HC group, *Prevotella copri* and *Phascolarctobacterium faecium* in the HT group, and *Klebsiella pneumoniae* in the HT-T2DM group. Co-occurrence network analysis indicates that *Prevotella copri*, *Mediterraneibacter gnavus*, *Alistipes onderdonkii* and some unidentified species act as key nodes in the network. Differentially functional pathway identified by PICRUSt2 were concentrated in nutrition and energy metabolism, as well as the biosynthesis of other secondary metabolites.

**Conclusions:**

Our study found significant differences in microbial community richness, composition, and function among the healthy controls, hypertension patients with and without T2DM. Some specific taxa may explain this difference and serve as potential therapeutic targets for hypertension, T2DM, and their coexistence.

## Introduction

Cardiovascular disease (CVD) is the leading cause of global mortality and about 17.9 million people die from it each year. According to the Global Burden of Disease (GBD) Study 2019, hypertension ranks first among modifiable risk factors that attribute to cardiovascular disorder burden [1]. Hypertension (HT) is the medical condition that blood pressures are too high when measured in two different days. With stronger force against artery walls, high blood pressure causes serious damage to arteries and plays important roles in the development of many cardiovascular diseases, including coronary heart disease, heart failure, and stroke events. So far, only 1 in 5 patients with hypertension have it under control worldwide and the treatment can be lifelong once diagnosed. Hypertension is prone to coexist with type 2 diabetes mellitus (T2DM), which is also an important risk factor for CVD. It is reported that the risk of diabetes increases 11% in hypertension patients in a propensity-score matching Chinese cohort and the risk of developing T2DM later for hypertensive patients may increase 2.646 times, though the risk drops to 8.3% after adjusting the propensity score [2]. However, the coexist of hypertension highly increased the incidence of coronary heart disease (CHD) and CHD mortality. As reported by a study included 49,775 Finnish subjects, the hazard ratio of female with serious hypertension (blood pressure ≥ 160/95 mmHg) only or T2DM only are 1.69 and 2.39, respectively, but is 3.31 for patients with both, comparing to male without either of these diseases. The corresponding hazard ratios for female are even higher, 2.37 and 5.63 for serious hypertension only or T2DM only, and 7.41 for both [3]. Another study by Pavlou et al. also reported that the coexistence of these two diseases may cause a four-fold increased risk in the development of cardiovascular disease [4]. Currently, there is no clear consensus on the association between hypertension and the risk of diabetes [2]. Hence, it is urgent to further illuminate the mechanisms behind hypertension and incident diabetes, as well as find out the potential therapeutic targets.

The importance of human microbiota has been revealed by the growing clinical and animal research in the homeostatic balance of host body [5]. Many studies have already demonstrated the altered microbiota composition in patients with digestive diseases, including inflammatory bowel diseases [6] and irritable bowel syndrome [7]. In recent years, studies are also focusing on the association between gut microbiota and immune regulation, lifestyle-related diseases and metabolism disorders. For examples, the altered microbiota profiles have been reported in HT and T2D patients [8, 9]. With the deep shotgun sequencing of the gut microbial DNA in 345 Chinese patients with T2DM, Qin et al. demonstrated that T2DM patients can be characterized by a changed microbiota profile with decreased abundance of butyrate-producing bacteria and increased opportunistic pathogens [10]. In addition, Tong et al. proved that the treatment of T2DM using metformin or herbal formula may ameliorate T2DM through changing gut microbiota structure and enriching beneficial bacteria, such as Blautia and Faecalibacterium spp [11]. Another research reported an increase in the abundance of phylum Firmicutes (p = 0.010) and Operational Taxonomic Units (OTUs) of Lactobacillus (p < 0.01) in individuals with known diabetes, accompanied by a graded decrease in butyrate-producing bacterial families such as Ruminococcaceae and Lachnospiraceae [12]. For the microbiota in HT patients, a decrease of short-chain fatty acid producers, such as Roseburia and Faecalibacterium prausnitzii, has been reported, while an increase of opportunistic pathogenic was also observed [13]. Li et al. proved that the microbiota profile in pre-hypertension patients is very similar to that in hypertension patients, and importantly reduced microbial abundance and diversity were observed in both groups [14]. However, currently no studies focused on the difference between hypertension patients with or without T2DM.

In this study, we recruited 72 hypertension patients without T2DM and 29 hypertension patients with T2DM, and acquired data of 40 healthy people from public database. We aimed to explore the differences of community structure and taxa abundance of gut microbiota between hypertension patients with or without T2DM, and between these 2 groups and healthy controls. Our studies may provide new insights and directions to identify specific bacteria associated with hypertension and incident diabetes and explain the potential mechanism behind the coexistence of these 2 diseases.

## Materials and methods

### Study population and samples

We enrolled 101 patients in this study (including 59 males and 42 females) from those who visit the Department of Cardiovascular Medicine at Shaoxing Second Hospital. Patients were further divided into two groups, hypertension with T2DM (HT-T2DM group n = 29) and hypertension without T2DM (HT group, n = 72). The diagnosis criteria of T2DM are plasma glucose ≥ 11.0 mmol/L at any time or fasting plasma glucose (FPG) level ≥ 7.0 mmol/L. Hypertension was diagnosed when systolic blood pressure ≥ 140 mm Hg and/or diastolic blood pressure ≥ 90 mm Hg in two different days. All the enrolled patients provided written informed consent before the recruitment and this study was approved by the Medical Ethics Committee of Shaoxing Second Hospital. In order to get a comprehensive understanding of the gut microbiota structure of these patients, we also download the 16 S rRNA gene sequencing data from another project in National Center for Biotechnology Information platform with the accession number PRJNA815750 (https://www.ncbi.nlm.nih.gov/bioproject/PRJNA815750). After age and BMI matching, a total of 40 samples were selected as the healthy control group for subsequent analysis.

### Clinical characteristic measurement

For each participant, their age, gender, body weight and height, systolic blood pressure, diastolic blood pressure and heart rate were recorded. All clinical tests were performed in the clinical pathology department of Shaoxing Second Hospital. The fasting venous blood samples of these patients were collected and preserved for further tests, including fasting blood glucose (FBG), total cholesterol (TC), triglycerides (TG), low-density lipoprotein cholesterol (LDLC), and high-density lipoprotein cholesterol (HDLC). The fecal samples were collected using sterile cryotubes and stored in -80℃ for further sequencing and analysis.

### DNA extraction and 16 S rRNA gene sequencing

We used the PowerMax Extraction Kit (MoBio Laboratories, Carlsbad, CA, USA) to extract the microbial genomic DNA in fecal samples based on the manufacture’s instruction. After that, agarose gel electrophoresis and a NanoDrop ND-1000 spectrophotometer (Thermo Fisher Scientific, Waltham, MA, USA) were applied to quantify the amount and purity of microbial DNA in stool samples. To amplify the16s V3 and V4 region, two universal primers were used, specifically 341 Forward Primer (5′-CCTACGGGAGGCAGCAG-3′) and 805 Reversed Primer (5′-GACTACHVGGGTWTCTAAT-3′). Polymerase Chain Reaction (PCR) was conducted in a 50 µL reaction system The cycle procedures for PCR were pre-denaturation at 98℃ for 30 s, followed by 25 cycles of denaturation at 98℃ for 15 s, annealing at 58℃ for 15 s, extension at 72℃ for 15 s, and a final extension at 72℃ for 1 min. AMPure XP Beads (Beckman Coulter, Indianapolis, IN) were then used to purify the products of PCR, while the concentration of DNA were determined by the PicoGreen double-stranded DNA Assay Kit (Invitrogen, Carlsbad, CA, USA). The DNA libraries were then sequenced on an Illumina NovaSeq 6000 pair-end 2 × 250 bp platform after quantity analysis at Shanghai Biotecan Pharmaceuticals Co., Ltd (Shanghai, China).

### Data processing, analysis and visualization

Qiime2 version 2023.2.0 [15] was employed to carry out DADA2 [16] processing of the raw sequencing data. Initially, data underwent quality filtering to remove adapter and barcode sequences, and trimming to an appropriate length to remove sequences with an average quality score below 25. Sequences were then dereplicated, evaluated for sequence variants, merged, and subjected to chimera checking through a standard DADA2 procedure. Any amplicon sequence variant (ASV) with a frequency less than 50 in all samples or appearing in fewer than three samples was filtered. Filtered representative sequences and biom-formatted tables were subsequently assigned with the Greengenes2 2022.10 database [17]. The resulting table and taxonomy artifacts were exported as a biom table and text file, respectively, for subsequent analyses following the addition of taxa data to the biom-formatted ASV table.

All alpha-diversity and beta-diversity indexes were computed and visualized using the “microeco” package (v0.15.0) [18]. The Principal Component Analysis (PCA), Principal Coordinate Analysis (PCoA), and Non-Metric Multidimensional Scaling (NMDS) plots based on Jaccard and Unweighted distances, the taxonomic composition bar plot, the feature abundance box plot, the Venn plot, and the heatmap plot were also generated by the “microeco” package. As the microbiota was presented in relative abundance, Linear Discriminant Analysis (LDA) effect size (LEfSe) analysis [19] was employed to compare differences in microbiota composition. After setting the alpha value to 0.01 and the LDA score threshold to 4, the LEfSe bar plot and corresponding cladogram were drawn using the “microeco” package. The co-occurrence network was calculated by the “microeco” packaged using Spearman analysis to compute correlation coefficients, and P-value threshold was set to 0.01, while the coefficient threshold was automatically optimized. The cluster_fast_greedy method was then employed for network clustering and visualization was conducted using Gephi (v0.10.1) [20]. The Phylogenetic Investigation of Communities by Reconstruction of Unobserved States (PICRUSt2) (v2.5.1) [21] workflow was applied for the prediction of metagenome functions of microbiota, and the functional pathways were annotated by Kyoto Encyclopedia of Genes and Genomes (KEGG) database [22–25].

### Statistical analysis

All the clinical characteristics were presented as average means ± standard deviation (SD) or numbers and percentages. The comparison clinical characteristics between different groups were conducted by Student’s *t*-test, Chi-square test and Fisher’s exact test using Python (version 3.10) and Benjamini-Hochberg FDR correction was applied to adjust p values in multiple test. P values < 0.05 were considered statistically significant. The Wilcoxon Rank Sum Test and Permutational multivariate analysis of variance (PerMANOVA) was used for testing the microbial alpha diversity and beta diversity difference between groups, respectively.

## Results

### The baseline information and taxonomy characteristics of the three groups

We recruited 72 hypertension without T2DM patients (HT group) and 29 hypertension with T2DM patients (HT-T2DM group) in this study. The baseline characteristics of our participants are listed in Table 1. The comparation are conducted between the patients with or without T2DM. After applying a Benjamini-Hochberg FDR correction, onlysignificant difference in GLU have been observed, with p value equal to 0.015, respectively. As the data for the HC group were obtained from a public database, only age and BMI information were available from their metadata. The average age of the HC group was 58.88 ± 5.15 years, while the average BMI was 21.14 ± 1.33. After annotating the representative sequences with gg2 database, the taxonomy information were collapsed into each level, and is shown in Table 2.

**Table 1:** Clinical characteristic of hypertension patients with or without T2DM

Characteristics	HT	HT-T2DM	Adjusted p value
gender			
Male	43	16	0.90
Female	29	13	
Smoking			
Yes	34	13	1.00
No	38	16	
Drinking			
Yes	40	18	0.97
No	32	11	
Age (years old)	64.10 ± 12.21	69.59 ± 11.94	0.1
BMI(kg/m2)	23.46 ± 3.52	25.40 ± 3.73	0.075
TG(mmol/L)	4.30 ± 1.07	4.17 ± 0.97	0.57
TC(mmol/L)	1.59 ± 1.18	1.64 ± 0.75	0.4
HDL-C(mmol/L)	1.13 ± 0.31	1.02 ± 0.25	0.19
LDL-C(mmol/L)	2.76 ± 0.85	2.62 ± 0.80	0.83
LP(a)(mmol/L)	153.08 ± 151.41	165.79 ± 141.71	0.92
ApoA-I(g/L)	1.23 ± 0.26	1.19 ± 0.19	0.84
ApoB(g/L)	0.81 ± 0.25	0.80 ± 0.20	0.92
Glucose(mmol/L)	5.33 ± 1.40	7.66 ± 2.20	< 0.001
SBP(mmHg)	142.40 ± 22.05	149.10 ± 23.77	0.24
DBP(mmHg)	80.5 ± 14.27	81.06 ± 12.29	0.99

Note: BMI: Body mass index; TG: Triglycerides; TC: Total cholesterol; HDL-C: High-density lipoprotein cholesterol; LDL-C: Low-density lipoprotein cholesterol; LP(a): Lipoprotein (a); ApoA-I: Apolipoprotein A-I; ApoB: Apolipoprotein B; SBP: Systolic Blood Pressure; DBP: diastolic blood pressure (DBP)

**Table 2:** A summary of different taxonomy levels

Index	HC	HT	HT-T2DM
Phylum	7.95 ± 1.61	7.18 ± 1.45	7.69 ± 1.05
Class	8.43 ± 1.74	7.60 ± 1.70	8.34 ± 1.62
Order	15.35 ± 3.06	13.47 ± 3.62	15.21 ± 3.57
Family	25.68 ± 6.42	21.88 ± 6.91	25.66 ± 7.63
Genus	59.55 ± 18.76	52.89 ± 20.63	58.41 ± 24.84
Species	87.85 ± 26.70	75.33 ± 29.29	85.41 ± 37.53

### The community composition and structure of the microbiome in HC, HT and HT-T2DM group

**Fig. 1:**
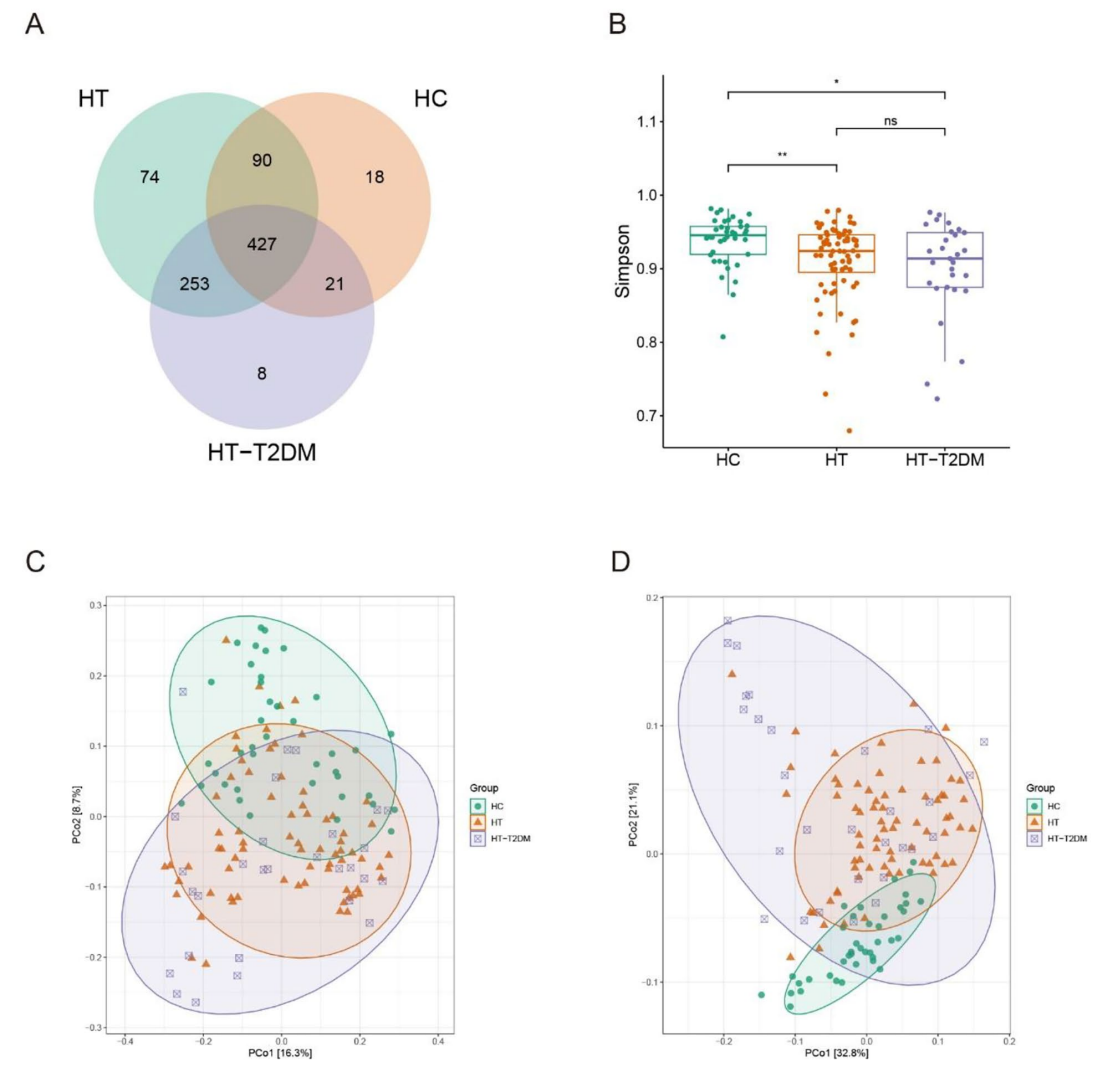
Overview of the microbiota structure. (**A**) Venn plot illustrating the unique and shared ASVs among the three groups. (**B**) Comparations of Simpson Index between the three groups. (**C**) and (**D**) Beta diversity between the three groups using Unweighted Unifrac PCoA and Weighted Unifrac PCoA, respectively

The 16 S rRNA gene sequencing data of these patients, along with the data of 40 health control downloaded from NCBI were compared and analyzed to illustrate the difference among these groups. Based on the Venn plot (Fig. 1A), the HC, HT, and HT-T2DM groups had 18, 74, and 8 unique ASVs, respectively, while 427 ASVs were found to be present in all three groups. The alpha and beta diversity analysis were performed to illustrate the microbial community richness and composition among the three groups. As shown in Fig. 1B, the Simpson index was significantly higher in the HC group compared to the HT and HT-T2DM groups, with p values equals to 0.0053 and 0.023, while no significant difference were observed for the Shannon index. Analysis of beta diversity using unweighted (Fig. 1C) and weighted (Fig. 1D) UniFrac distances both demonstrated significant differences in distance, with p-values of < 0.001 for the comparisons between each group. The composition of the HC group microbiota community was clearly distinct from those of the HT and HT-T2DM groups, indicating a significant difference in microbiota composition between the HC and HT/HT-T2DM groups.

**Fig. 2:**
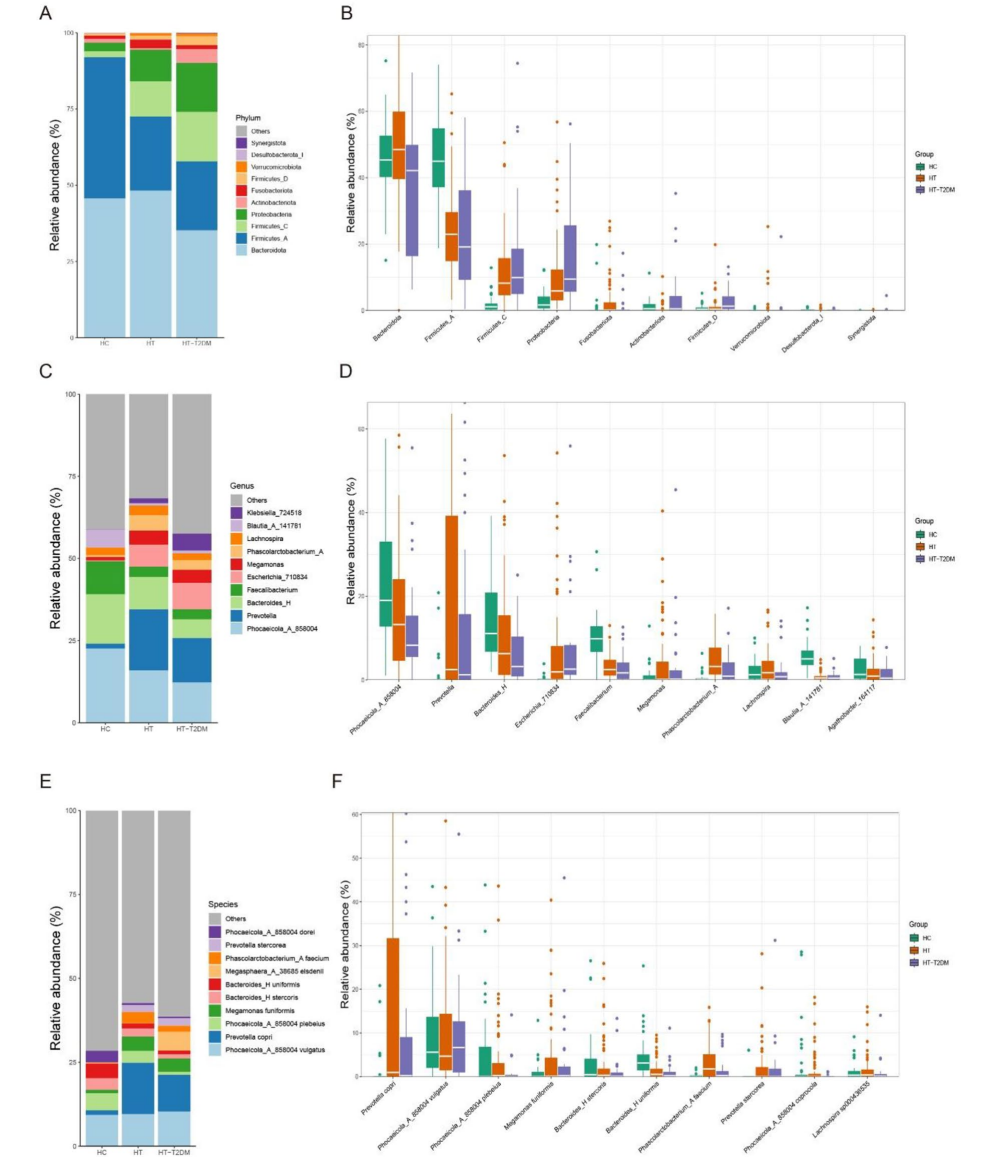
Bar plot and box plot of the top 10 most abundant species at different levels. (**A**) and (**B**) Phylum level. (**C**) and (**D**) Genus level. (**E**) and (**F**) Species level

Figure 2 illustrates the microbial composition at different taxonomic levels. The top 4 abundant phyla in HT and HT-T2DM group are Bacteroidetes, Firmicutes_A, Firmicutes_C and Proteobacteria. At the genus level, the HC group had a higher abundance of *Blautia* but lower abundances of *Prevotella* and *Klebsiella* compared to the HT and HT-T2DM groups. At the species level, the HC group had a higher abundance of Phocaeocola dorel and Bacteroides uniformis but lower abundances of Prevotella stercorea and Phascolarctobacterium faecium compared to the other groups. Additionally, the HT-T2DM group had a higher abundance of Megasphaera elsdenii.

### LDA analysis reveals the phylogenetic and taxonomic profiles in microbiome among the three groups

**Fig. 3:**
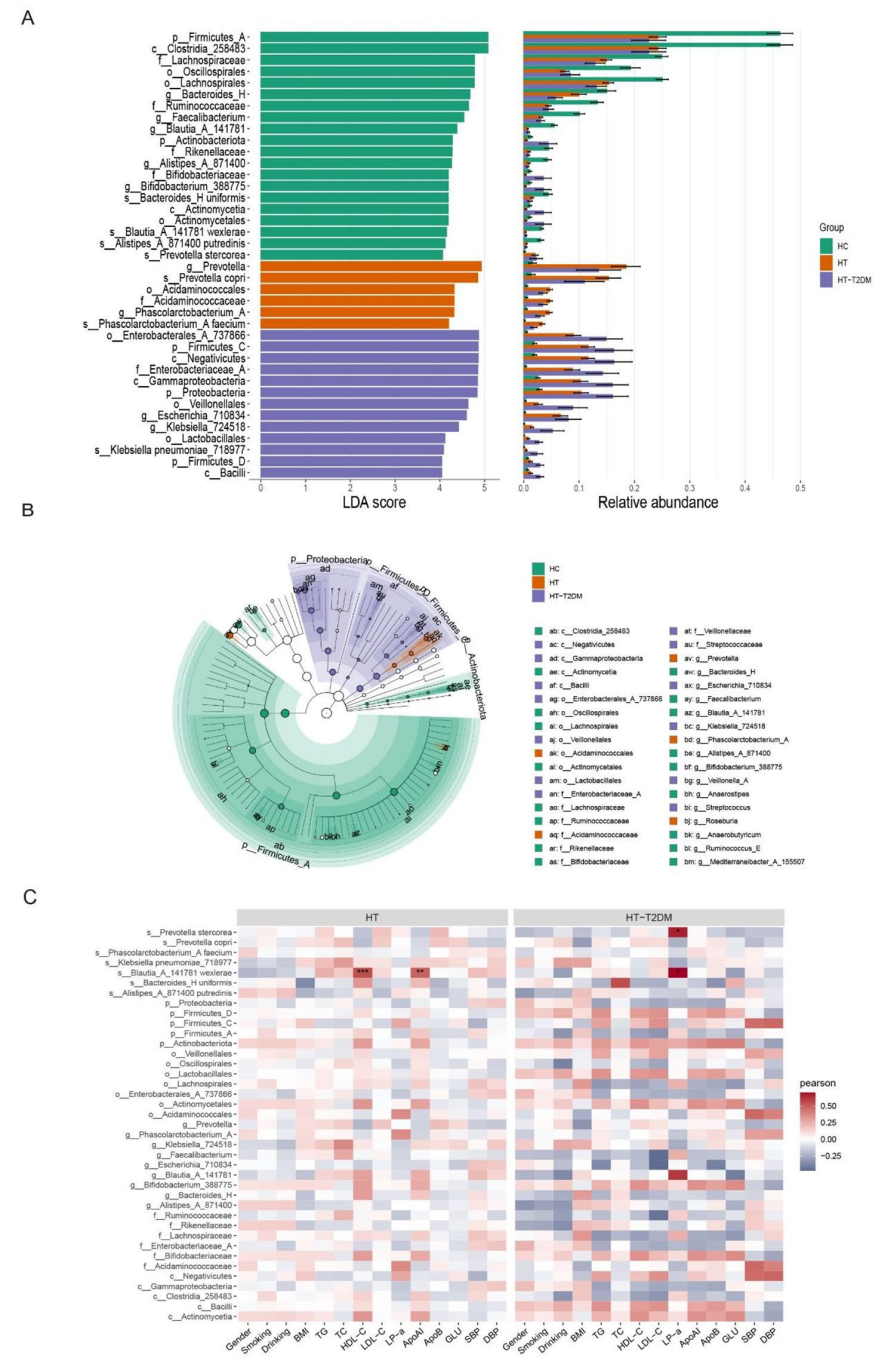
LEfSe analysis and correlation between differentially abundant species and clinical features. (**A**) Gut microbiota difference between the three groups were identified with a LEfSe analysis with LDA score threshold > 4.0. (**B**) The cladogram plot. (**C**) The correlations between clinical characteristics and differentially abundant ASVs identified by LEfSe analysis

Next, we want to find the difference in certain taxa among HC, HT and HT-T2DM groups. Hence, we performed LEfSe analysis, using effect size measurement to enrich the bacterial taxa with different abundance among the three groups. With the threshold of significance (p < 0.05) and LDA score > 4, taxon with different abundance are displayed in Fig. 3A. At Species level, *Bacteroides uniformis*, *Blautia wexlerae*, *Alistipes putredinis* and *Prevotella stercorea* in HC group, *Prevotella copri, and Phascolarctobacterium faecium* in HT group, and *Klebsiella pneumoniae* in HT-T2DM group were found to have significantly high impact on the difference among these three groups. We also generated cladograms from the LEfSe analysis to offer a visual result of the phylogenetic distribution of these samples, from class to genus level. The size of each circle in the cladogram represents the abundance of certain taxa (Fig. 3B). We further analyzed the correlation between the differentially abundant species obtained from the LEfSe analysis and clinical features. In the HT group, the abundance of *Blautia wexlerae* was significantly positively correlated with HDL-C (p < 0.001) and ApoAI (p = 0.007). In the HT-T2DM group, the abundance of *Blautia wexlerae* and *Prevotella stercorea* were both significantly positively correlated with LP-a (p = 0.011 and p = 0.032, respectively) (Fig. 3C).

### The co-occurrence network of the three groups

**Fig. 4:**
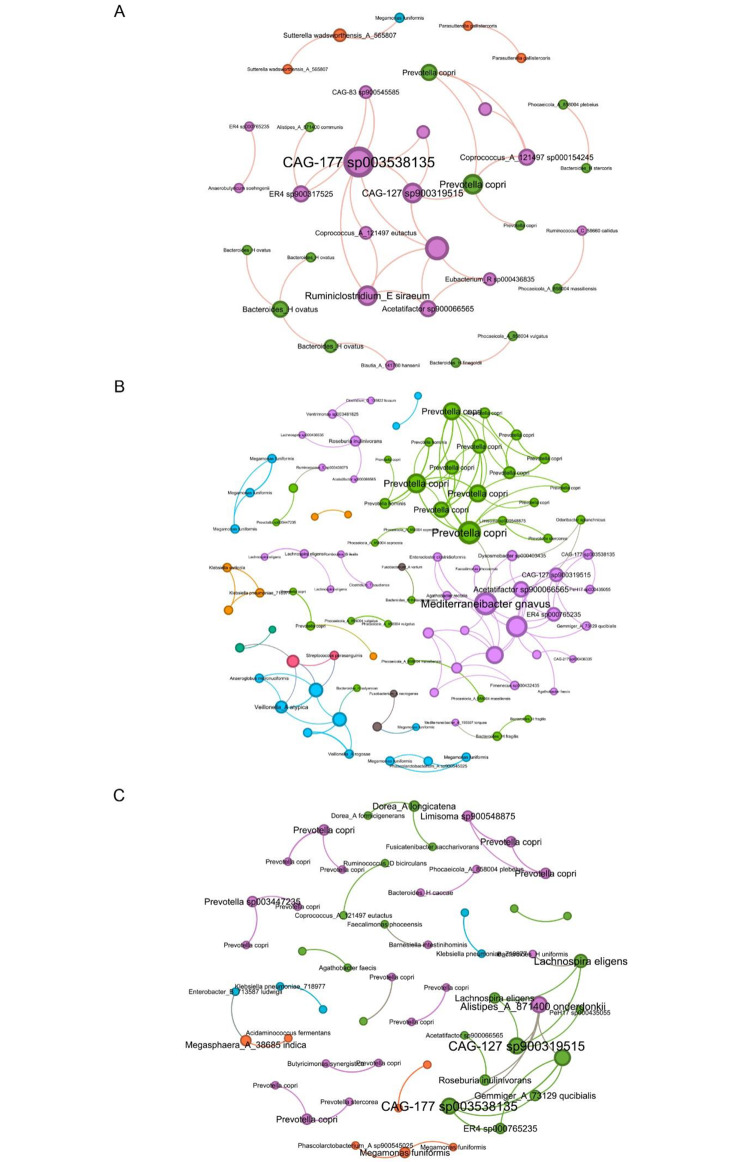
Co-occurrence network analysis for the three groups. (**A**) HC group. (**B**) HT group. (**C**) HT-T2DM group. Node size was presented by its degree (5–15). The node colors were based on phylum level. The text in the figure labels the nodes with species-level information

To further explore keystone taxa among the three groups, we constructed microbial co-occurrence networks using Spearman correlations between different taxa (Fig. 4), and the summary of the co-occurrence networks in the three groups were list in Table 3. All three networks showed a close association between Firmicutes_A and Bacteroidota, with this interaction being more evident and complex in the HT group. Some specific species act as key nodes in the network. For example, in the HC group, an unidentified species with placeholder CAG-177 sp003538135 under Acutalibacteraceae family, an unidentified species with placeholder CAG-127 sp900319515 under Lachnospiraceae were important nodes that were connected to *Prevotella copri*. In the HT group, *Prevotella copri* was linked to *Mediterraneibacter gnavus*. In the HT-T2DM group, CAG-177 sp003538135 and CAG-127 sp900319515 were found to be associated with *Alistipes onderdonkii*.

**Table 3:** Summary of the co-occurrence networks in the three groups

Group	Vertex	Edge	Average Degree	Average Path Length	Positive Label	Negative Label
HC	34	35	2.06	1.70	100%	0%
HT	94	120	2.55	1.69	92.5%	7.5%
HT-T2DM	55	41	1.49	1.59	100%	0%

### Difference of microbial metabolic pathways in the three groups

**Fig. 5:**
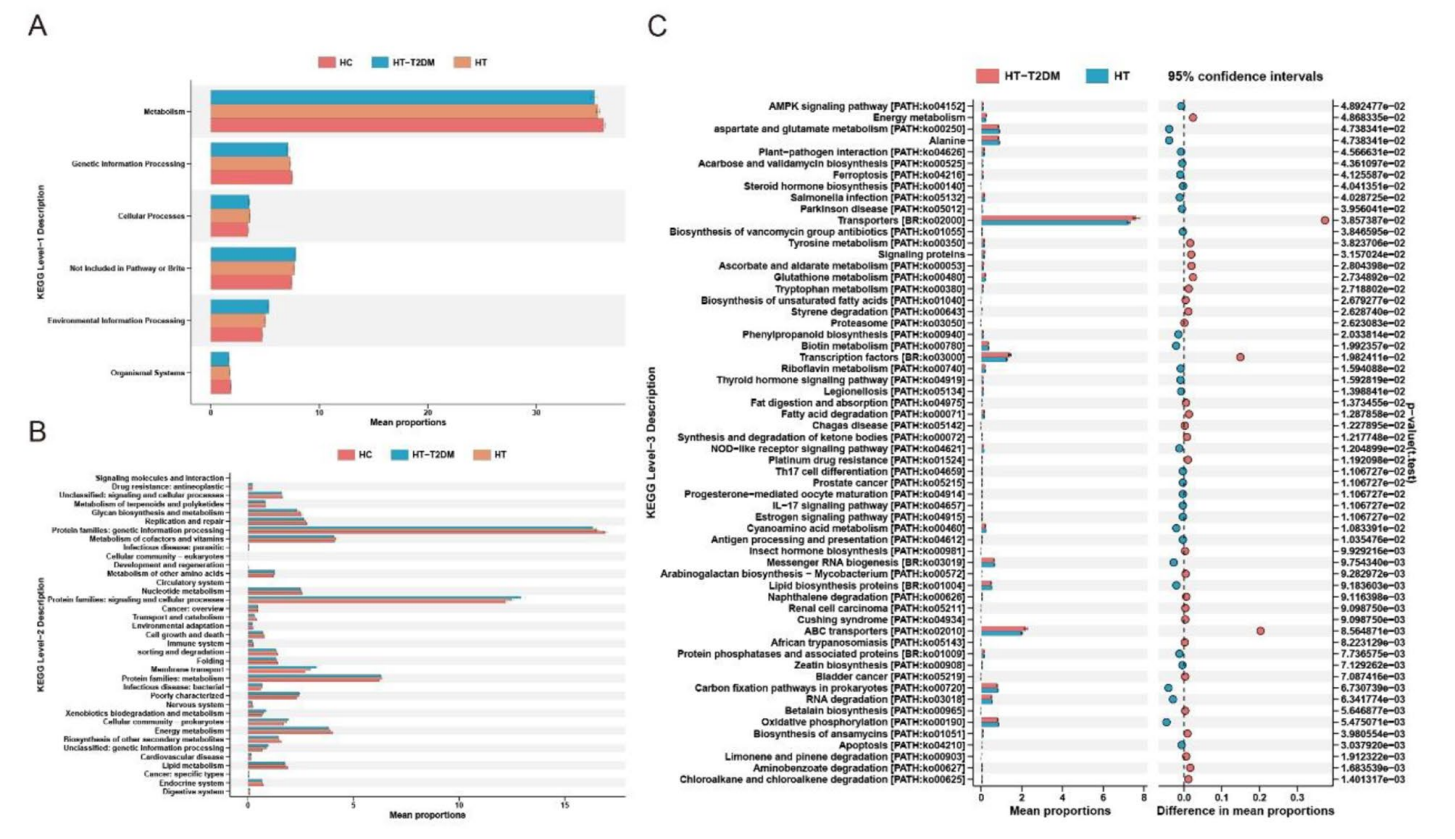
The results of KEGG annotations by PICRUSt2-predicted based on 16 S rRNA gene sequencing data of the three groups. (**A**) and (**B**) KEGG level 1 and level 2 annotation for the differently abundant function pathway in the three groups. (**C**) KEGG level 3 annotation for the differently abundant function pathway between HT and HT-T2DM group

PICRUSt 2 was used for predicting functional abundances, and the Kyoto Encyclopedia of Genes and Genomes (KEGG) database was subsequently used for functional pathway annotation. As shown in Fig. 5, we compared the differences in functional pathways among the three groups at KEGG levels 1 and 2. Compared to the HC group, the HT and HT-T2DM groups had lower metabolism functions at KEGG level 1. According to the results at KEGG level 2, lipid metabolism, biosynthesis of other secondary metabolites, and energy metabolism were also lower in the HT and HT-T2DM groups than in the HC group. However, due to the large number of functional pathways involved at level 3, we only compared the functional pathways between the HT and HT-T2DM groups, and The majority of the different metabolic pathways were concentrated in nutrition and energy metabolism.

## Discussion

Hypertension is the top modifiable risk factors that attribute to cardiovascular disorder burden. Coexisted with type 2 diabetes mellitus, hypertension may cause serious damage to arteries and plays important roles in the development of many cardiovascular diseases, including coronary heart disease, heart failure, and stroke events. Therefore, it is urgent to illuminate the mechanisms behind hypertension and incident diabetes. In this study, we compared the microbiota profile in hypertension patients with and without T2DM, and separately compared these two groups with health control. The beta diversity analysis showed significant difference in the Bracurtis distance and UniFrac distance and a significant difference of alpha diversity, which is consistent with many previous studies [8, 9].

The LEfSe analysis, identifies *Prevotella copri, and Phascolarctobacterium faecium* in HT group, and *Klebsiella pneumoniae* in HT-T2DM group have significantly high impact on the taxonomy difference. According to the study by Li et al., hypertensive individuals had decreased microbial richness and diversity, Prevotella-dominated gut enterotype, and overgrowth of bacteria such as *Prevotella* and *Klebsiella* [14]. Another research also reported the correlation between *Prevotella* and elevated blood pressure [26]. However, currently there’s no research on *Preotella copri* in hypertension patients, but it may play an essential role in triggering the inflammatory response in disease such as rheumatoid arthritis [27]. There is increasing evidence that inflammation contributes to the pathogenesis of hypertension, while an ecological imbalance in the intestinal epithelial barrier can lead to systemic inflammation and impair intestinal mechanical transmission. These changes activate mechanisms that are traditionally associated with blood pressure regulation, such as the renin-angiotensin-aldosterone system, the autonomic nervous system, and the immune system [28, 29]. Some studies reported the enrichment of gut *Klebsiella pneumoniae* in hypertension patients [30], but now directly evidence is found between and *Klebsiella pneumoniae* T2DM. Further research is needed to determine whether Klebsiella pneumoniae is a cause of T2DM or a result of gut microbiota dysbiosis. In the HC group, *Blautia wexlerae* was found to have a significantly high impact on the taxonomy difference, and in the correlation analysis between microbiota and clinical features, it showed a significant positive correlation with HDL-C, ApoAI, and LP-a. Based on the Hosomi’s research, *Blautia wexlera* has been found to be negatively correlated with T2DM and obesity, and may play a role through multiple unique amino acid metabolism pathways [31], suggesting that it may serve as a therapeutic target for T2DM.

Still, there are some limitations in this study. The sample size, especially in the HT-T2DM group, made more stringent methods such as 1:1 matching propensity score analysis are not suitable for eliminating covariates in our study. In addition, the HC group represented healthy individuals based on public database data. The composition of microbiota is affected by various factors including but not limited to age, region, dietary habits, and intestinal type. Since our study focused on hypertensive patients, who are typically older, finding age-matched healthy controls from a similar geographic region proved challenging. Despite our best efforts to match most information of healthy controls, the 40 selected controls still had a lower total sequencing reads number (50k raw reads) than ours (100k raw reads). We used a strategy based on the minimum ASV count for rarefaction, but this may not completely eliminate the influence of microbiota information. Therefore, in future studies, efforts should be made to recruit more matched controls and expand the sample size to eliminate the influence of numerous covariates. Additionally, we only sequenced the 16 S V3-V4 region, so although we used the latest Greengene2 database to obtain classification at the species level as much as possible, more than 50% of species could not be identified. Nanopore-based full-length 16 S sequencing or metagenomic sequencing would be advantageous for obtaining more comprehensive species information.

In conclusion, the intestinal flora was compared between hypertension patients with or without T2DM, including relative changes in the abundance of intestinal microorganism. As far as we know, no one reported the influence of microbiota in HT with T2DM, and our findings may help to develop novel therapeutic target for prevention, control or even treatment for hypertension, T2DM and the coexistence of these two diseases.

## Data Availability

The raw sequencing data are stored in NCBI with BioProject ID PRJNA815750. Other data that are analyzed during the current study are available from the corresponding author on reasonable request.
